# Acetabular fractures following rugby tackles: a case series

**DOI:** 10.1186/1752-1947-5-505

**Published:** 2011-10-05

**Authors:** Daniel W Good, Michael Leonard, Darren Lui, Seamus Morris, John P McElwain

**Affiliations:** 1 Department of Trauma Orthopaedics and Reconstructive Pelvic and Acetabular Surgery, Adelaide and Meath Incorporating the National Childrens Hospital, Tallaght, Dublin 24, Ireland

## Abstract

**Introduction:**

Rugby is the third most popular team contact sport in the world and is increasing in popularity. In 1995, rugby in Europe turned professional, and with this has come an increased rate of injury.

**Case presentation:**

In a six-month period from July to December, two open reduction and internal fixations of acetabular fractures were performed in young Caucasian men (16 and 24 years old) who sustained their injuries after rugby tackles. Both of these cases are described as well as the biomechanical factors contributing to the fracture and the recovery. Acetabular fractures of the hip during sport are rare occurrences.

**Conclusion:**

Our recent experience of two cases over a six-month period creates concern that these high-energy injuries may become more frequent as rugby continues to adopt advanced training regimens. Protective equipment is unlikely to reduce the forces imparted across the hip joint; however, limiting 'the tackle' to only two players may well reduce the likelihood of this life-altering injury.

## Introduction

Rugby is the third most popular team contact sport in the world and is increasing in popularity [1]. Rugby Union underwent a major change in 1995 when the sport turned professional. With this also came an increased rate of injury [1]. Numerous studies have identified an increase in the rates of injury during both professional and amateur rugby in recent years [1-4]. Garraway *et al. *[2] suggested that this increase in injury incidence was due to an increased emphasis on speed, strength and stamina.

Acetabular fractures are an uncommon injury with an incidence of approximately three per 100,000 population [5]. These fractures occur as a result of high-velocity trauma such as road traffic accidents, particularly in younger patients, and are associated with significant morbidity and mortality [6], including sciatic nerve injury and early post-traumatic arthritis. Acetabular fractures from sport are extremely rare, and we describe two cases which occurred during rugby union.

In a six-month period from July to December, two open reduction and internal fixations of acetabular fractures were performed in young Caucasian men (16 and 24 years old) who sustained their injuries after rugby tackles. Both of these cases are described below.

## Case presentations

### Case 1

**Figure 1:**
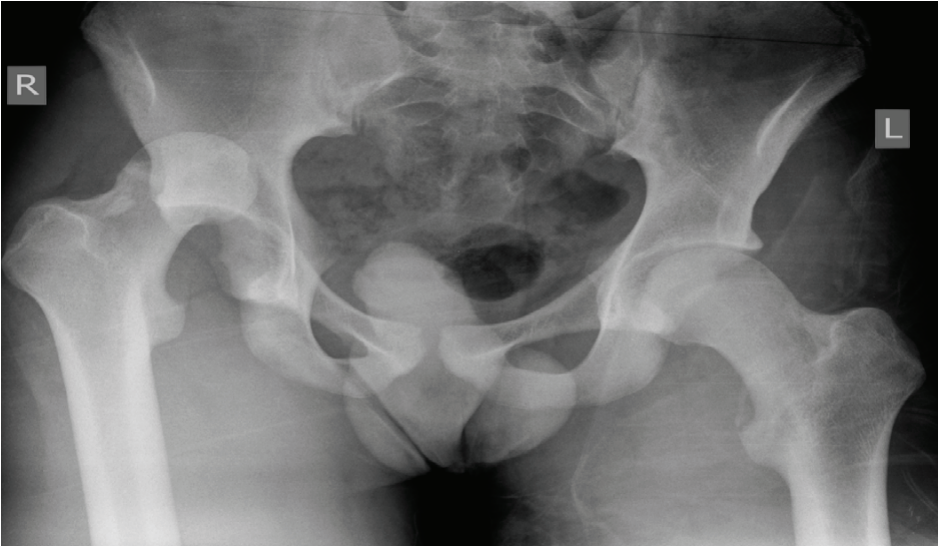
**Fracture-dislocation of right hip (Case 1)**.

**Figure 2:**
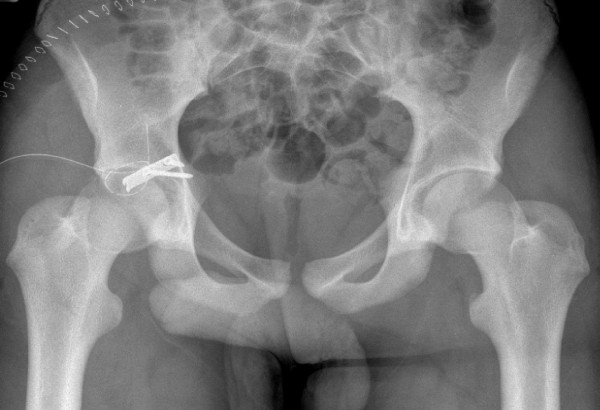
**Postoperative X-ray (Case 1)**.

The first case was a 16-year-old, male Caucasian, weighing 60 kg (body mass index (BMI) = 20.5), who incurred his injury playing school rugby. Running with the ball, he was tackled first from his left causing him to stumble to his right. He was then tackled by another player from his left, falling onto his flexed right knee. He felt immediate pain and he was unable to move his right leg. He was taken to his local hospital where images of his pelvis revealed a posterior fracture-dislocation of his right hip joint (Figure 1). This was reduced interoperatively within two hours. Postoperatively he was transferred to our institution for definitive management. A computerized tomography (CT) scan of his pelvis demonstrated a displaced fragment of the posterior wall of his acetabulum and an examination under anesthesia revealed instability of the joint. We elected to undertake open reduction and internal fixation. A posterior Kocher-Langenbach approach was performed and the posterior wall fragment was reduced and fixed with a two-hole spring plate (Figure 2). He underwent an uneventful recovery and was discharged on the third postoperative day. He remained non-weight bearing on his right leg with crutches for six weeks, with subsequent progression to full weight bearing. On review six months after the operation, there was union demonstrated on X-ray. Our patient was pain free, fully weight bearing and undergoing light training. He will be kept under long-term review.

### Case 2

**Figure 3:**
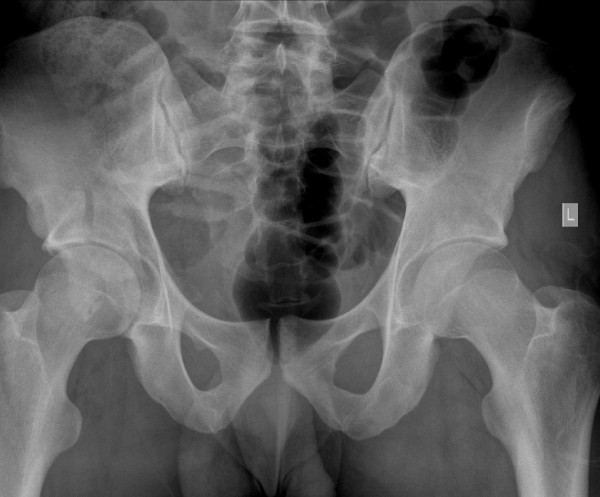
**Comminuted fracture of right acetabulum (Case 2)**.

**Figure 4:**
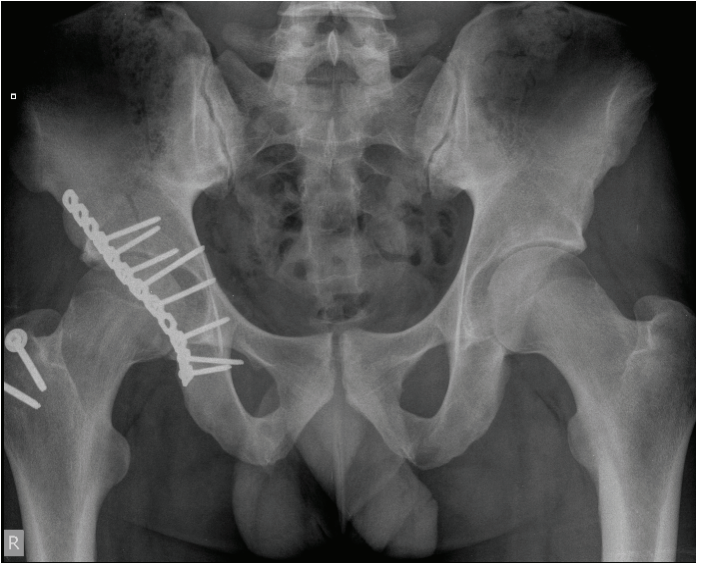
**Postoperative X-ray (Case 2)**.

This patient was a 24-year-old Caucasian man, weighing 105 kg (BMI = 26), playing amateur rugby at a high standard. During open play, whilst running, he was tackled from his left side causing him to stumble to his right. With his right leg planted he was tackled by another player from his left causing him to land on his flexed right knee. He felt immediate pain and was unable to bear weight. He was taken to his local hospital where imaging of his pelvis showed a comminuted posterior wall-posterior column right acetabular fracture (Figure 3). He went on to have a CT scan of his pelvis which confirmed the plain film findings and also demonstrated marginal impaction of his articular surface, a recognized poor prognostic indicator. He was transferred to our institution for definitive management and underwent open reduction and internal fixation through a posterior Kocher-Langenbach approach (Figure 4). His articular surface was elevated and supported with a local bone graft from his greater trochanter. His postoperative recovery was uneventful and he was discharged on the third postoperative day. He remained non-weight bearing on his right leg with crutches for six weeks, with subsequent progression to full weight bearing. Six months postoperatively, our patient was doing well, fully weight bearing, doing light gym work and showed union on X-ray. He will be kept under long-term review.

## Discussion

These two cases show clearly how rugby, even at amateur level, is a sport that imparts high energy. The two injuries would normally be seen following high-speed motor vehicle accidents.

Professionalism has made rugby players fitter, heavier and honed their ability to make a 'big hit' tackle. Training routines are designed for this purpose. Professional coaching methods are being applied to amateur teams and have increased injuries at this level [2]. An International Rugby Board study of the 2003 World Cup showed that injuries had increased, which was also due to players having a higher BMI and a 30% increase in the time the ball was in play [7].

Rugby involves four phases of play: open play, the tackle, the ruck and maul and set pieces. Most injuries in rugby occur during the tackle phase (36% to 56%) [7-9]. The tackled player has twice the incidence of injury than the tackler [7], with one-third of injuries occurring when there is a difference in tackling speeds [7] (the lower momentum player having four times the injury incidence [9]). This is mirrored in the amateur game [10,11]. Studies show that players with a higher BMI have higher injury rates [10].

The literature is consistent on the types and frequency of rugby injuries. Soft tissue injuries account for approximately 50% of all injuries [7-9,12]. The lower limb is most frequently affected by injury and accounts for 42% to 55% of all injuries [8,9]. Hip injuries account for only 2% of injuries to the lower limb, with the thigh (19%), knee (20%), ankle (6%) and foot (3.5%) all accounting for more [8].

These two cases share a common mechanism of injury; this involved a fall onto a flexed knee resulting from a 'double tackle' whereby the player is tackled by two opposing players. Letournel and Judet [13] showed that the posterior rim of the acetabulum bears the impact from the femoral head in this leg position. Acetabular fractures from sports are a rare occurrence and cases have often involved the same mechanism of injury as in our case [14,15]. Joint reactive force (JRF) is involved in hip joint biomechanics and represents the sum of the mechanical forces acting across the hip joint. During walking, JRF is approximately 2.5 × body weight (BW), 4.8 × BW during jogging and 8 × BW during stumbling [16]. The JRF in these cases is likely to have been much higher with the added force from a 'double tackle' whilst stumbling. It is no coincidence that the more severe fracture was in case 2 where the weight of our patient and tacklers was far higher, leading to a higher JRF targeted at his posterior rim. Studies have shown that there is an increased contact area of the femoral head on the acetabulum with increasing loads [17,18]. This is demonstrated in our cases: the injury in case 1 occurred during under-16 school rugby where player weights are lower than in case 2 (adult rugby). The energy (load) in case 1 was lower than case 2 and resulted in a smaller contact area against the posterior rim, and therefore a smaller fracture fragment compared with the fracture seen in case 2.

Our recent experience causes concern that these injuries are likely to become more frequent as rugby continues to adopt advanced training regimens and players become heavier. Acetabular fractures in such a young population carries with it significant morbidity, in the form of avascular necrosis, sciatic nerve injury and, in particular, early post-traumatic arthritis which may require a total hip replacement. The prognosis for the two young men in this series remains guarded; case 1 involved a fracture-dislocation and case 2 involved marginal impaction, both of which are associated with poor long-term outcome. There are also biomechanical factors present in these two cases which increase their risk of post-traumatic arthritis. These factors include intra-articular contact and pressure, loss of congruence and stiffness of the fracture fixation. In posterior wall fractures of the acetabulum, the greatest change in the contact area between the acetabulum and the femoral head are seen in the smallest of defects [19]. There is evidence that an increased contact area leads to higher stress in the joint cartilage, which can lead to a cascade of degenerative changes and develop into arthritis [20,21]. Cadaveric studies of posterior wall fracture patterns have also shown that there is a change in the contact pattern from a uniform contact area to one of increased contact area and peak pressures in the superior aspect of the acetabulum [21]. This is also associated with decreased pressures in the anterior and posterior walls [21]. This all leads to an increased risk of post-traumatic arthritis in both our patients.

## Conclusion

Rugby's new professionalism has resulted in improved training techniques that have been adopted by amateurs, resulting in fitter, heavier players and also an emphasis on 'the big hit' during open play. These two cases illustrate that rugby is now clearly a high-energy impact sport. The resulting fractures in our two cases were similar in mechanism of injury to other reported cases of acetabular fractures during sports [14,15]. A key factor in their injury was that both cases involved a 'double tackle'. This likely led to a large increase in the JRF and contributed to their fractures. Protective equipment is unlikely to compensate for this additional JRF, however limiting the tackle to only two players, the tackler and tackled player, may well reduce the likelihood of these life-altering injuries.

## Consent

Written and informed consent was obtained from the patient and legal guardian in case 1, and the patient in case 2, for publication of these cases and any accompanying images.

## Competing interests

The authors declare that they have no competing interests.

## Authors' contributions

DG was heavily involved in all aspects of the case report, from data collection, writing the manuscript, editing and final approval. ML had the initial idea for the case report and was heavily involved in the writing and editing of the manuscript. DL was involved in the data collection and editing of the manuscript. SM was involved in the editing of the manuscript, including discussion topics and final approval of the manuscript. JPM was involved in the editing and final approval of the manuscript. All authors read and approved the final manuscript.
